# Inferential Emotion Tracking reveals impaired context-based emotion processing in individuals with high Autism Quotient scores

**DOI:** 10.1038/s41598-023-35371-6

**Published:** 2023-05-19

**Authors:** Jefferson Ortega, Zhimin Chen, David Whitney

**Affiliations:** 1 Department of Psychology, University of California, Berkeley, CA 94720 USA; 2 Vision Science Program, University of California, Berkeley, CA 94720 USA; 3 Helen Wills Neuroscience Institute, University of California, Berkeley, CA 94720 USA

**Keywords:** Perception, Human behaviour

## Abstract

Emotion perception is essential for successful social interactions and maintaining long-term relationships with friends and family. Individuals with autism spectrum disorder (ASD) experience social communication deficits and have reported difficulties in facial expression recognition. However, emotion recognition depends on more than just processing face expression; context is critically important to correctly infer the emotions of others. Whether context-based emotion processing is impacted in those with Autism remains unclear. Here, we used a recently developed context-based emotion perception task, called Inferential Emotion Tracking (IET), and investigated whether individuals who scored high on the Autism Spectrum Quotient (AQ) had deficits in context-based emotion perception. Using 34 videos (including Hollywood movies, home videos, and documentaries), we tested 102 participants as they continuously tracked the affect (valence and arousal) of a blurred-out, invisible character. We found that individual differences in Autism Quotient scores were more strongly correlated with IET task accuracy than they are with traditional face emotion perception tasks. This correlation remained significant even when controlling for potential covarying factors, general intelligence, and performance on traditional face perception tasks. These findings suggest that individuals with ASD may have impaired perception of contextual information, it reveals the importance of developing ecologically relevant emotion perception tasks in order to better assess and treat ASD, and it provides a new direction for further research on context-based emotion perception deficits in ASD.

## Introduction

Emotion recognition and processing are essential for successful social interactions. Emotions play an important role in our social lives and in our understanding of others, and thus, shape the way that we understand the world around us. For example, individuals with autism spectrum disorder (ASD) have reported impairments in facial expression recognition, which could have knock-on consequences for other perceptual and social functions and could be a contributing factor in the reported deficits in social communication^1,2^. ASD is a neurodevelopmental disorder with an early onset and is characterized by impairments in social interaction and repetitive behaviors^2^. These deficits have often been attributed to an impairment in Theory of Mind, which is the ability to infer the mental states of others^3–5^.

One popular measure of Theory of Mind is the Reading the Mind in the Eyes Test^6^, also known as the Eyes Test, which requires participants to infer the emotion of a person based on their eyes alone, without any other information about the face or context. In this task, participants choose, among a selection of mental states, a single emotion label that they believe reflects the expression in the pair of isolated eyes. The Eyes Test distinguishes between typical controls and individuals with ASD^6–9^: performance on the Eyes Test is lower in individuals diagnosed with ASD compared to controls^7^ and it is correlated with Autism Spectrum Quotient (AQ) scores^6^. However, the Eyes Test has also been criticized on several grounds: it does not mimic how emotion is experienced in the real world; it lacks spatial context that is naturally experienced alongside facial expressions^10–14^; and it lacks temporal context, including how emotions change over time and how recent events influence an individual’s current emotional state ^15,16^. Moreover, some studies have found no difference in performance on the Eyes Test between individuals with ASD and other disorders like schizophrenia^17^ and alexithymia^18^. Additionally, some researchers argue that Theory of Mind, as operationalized by the Eyes Test, is not the key component of the underlying deficit in social communication that is normally observed in ASD. Instead, they suggest that ASD is not characterized by a single cognitive impairment but a deficit in a collection of higher-order cognitive abilities^19–24^. Specifically, alternative theories suggest that there are deficits in meta-learning^19^ and deficits in updating priors^23^ in individuals with ASD.

Despite the debate over the Eyes Test, it is well known that individuals with ASD have emotion perception deficits^1–3,25–30^, and this is true especially in more ecologically valid and dynamic situations^31–33^. These deficits have been attributed to moderators like emotion complexity^34^ and abnormal holistic processing of faces^30,35–37^ (though more recent studies suggest that individuals with ASD are able to utilize holistic face processing^38–40^). In addition, previous studies have also found that individuals with ASD display impairments in daily tasks that involve social interaction,^41,42^ even when there may be no impairment in contrived, less ecologically-valid experimental tasks of social cognition. These findings raise the possibility that the current assessments of social cognition in ASD may lack some critical attributes that are normally experienced during everyday social interactions^10,11,13^. In particular, existing popular tests^6,43,44^ do not measure or capture the role of spatial and temporal context in emotion perception.

Contextual information is critical for emotion perception. It influences emotion perception even at the early stages of face processing^45,46^, it is unintentionally and effortlessly integrated with facial expressions^47^, and it is an integral part of emotion perception in the real world^13,14^. More surprisingly, observers have been found to accurately and rapidly infer the emotions of characters in a scene without access to facial expressions, while using only contextual information^10–12^. The idea that “contextual blindness” may be a key component in ASD has been discussed before^48^ and has been attributed to the weak central coherence hypothesis^49^. Central coherence is the ability to combine individual pieces of information together into a coherent whole and has been suggested to be a key problem in ASD^49^. Weak central coherence in ASD could manifest as a reduced ability to integrate contextual information with face and body information when inferring the emotions of people in the real world. Previous research has found that individuals with ASD are less able to use contextual cues to infer the emotions of blurred-out faces^50^ supporting the idea that weak central coherence may affect how individuals with ASD integrate emotional cues in the real world.

Contextual information is not only present in the spatial properties of background scenes (e.g., background environment, scene information, surrounding faces and bodies, etc.), but there is also a temporal context which involves the integration of social information over time. Temporal context refers to the idea that information about emotions is dynamic, unfolds over time, and is subject to change^15,51,52^. Both spatial and temporal context can be informative in emotion perception. One example of temporal context relates to noticing when the emotion of another individual has changed. For example, when having a conversation with a friend, if you were to say something that offends them, then their emotion will change depending on the intensity of the offense. To successfully navigate the conversation, you would need to have noticed that the emotion of your friend has changed, in a timely manner, and either apologize or change the topic of conversation. Individuals with ASD may be impaired in such circumstances, because they have impairments processing dynamic complex scenes^53^. Moreover, many studies have reported differences in the processing of temporal context in typical individuals compared to those with ASD^54–57^. However, there is currently a dearth of research on how emotion perception of dynamic stimuli, which includes natural spatial context, is affected in individuals with ASD.

In the present study, we investigated whether individuals who scored high on the Autism Quotient^58^ (AQ) have an impaired ability to infer the emotion of a blurred-out (invisible) character using dynamic contextual information. To investigate this, we recruited a fairly large sample (*n* = 102) and had participants complete an Inferential Emotion Tracking^10,11^ (IET) task, where participants used a 2D valence-arousal rating grid to continuously track the emotion of a blurred-out (invisible) character while watching a series of short (1–3 min) movie clips. Each participant watched and rated a total of 35 different movie clips (which included Hollywood movies, documentaries, and home videos), and then completed a battery of questionnaires at the end of the experiment. To foreshadow the results, we found that individual differences on the IET task correlated strongly with AQ scores, suggesting that context-based emotion perception may be impaired in ASD, while the correlation between participants' scores on the Eyes Test and on the AQ questionnaire was not significant.

## Results

All analysis scripts and datasets are available at the Open Science Framework (https://osf.io/zku24/). All statistical analyses were performed using Python.

### IET task performance

**Figure 1:**
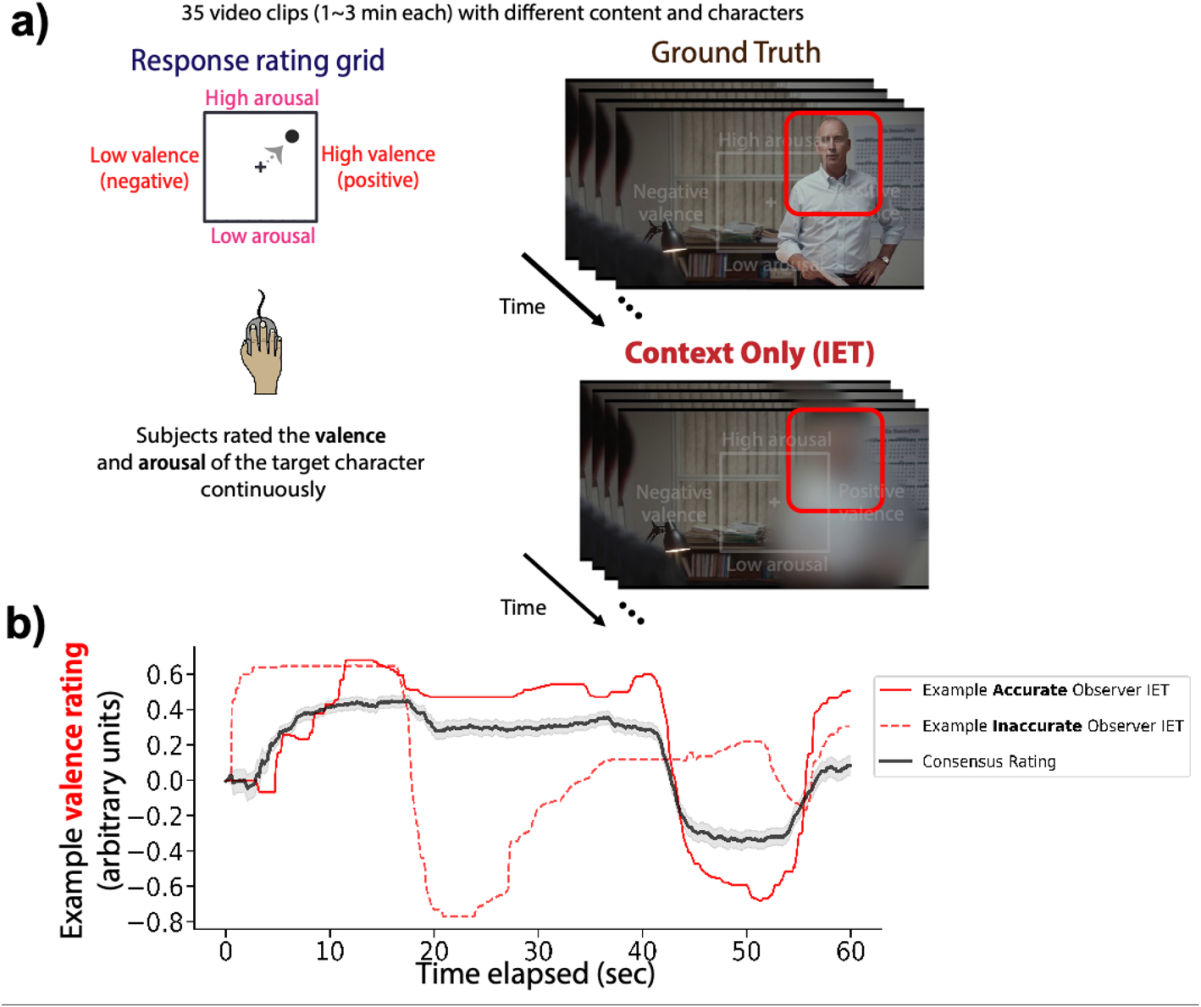
Inferential Emotion Tracking (IET) task paradigm. (**a**) One hundred and two participants rated a total of 35 different video clips, which included Hollywood movies, documentaries, and home videos. A 2D valence-arousal rating grid was superimposed on the video and participants were required to rate the emotion of the target character. The red outline indicating the target character for a given trial was only shown on a single frame before the start of the trial. (**b**) An example of an accurate observer (solid red line) and an inaccurate observer (dashed red line) compared to the averaged ratings (consensus rating) of the context only condition (black line). Shaded regions on the consensus rating represent 1 standard error of the mean. Videos shown in this figure and study are publicly available (https://osf.io/f9rxn).

**Figure 2:**
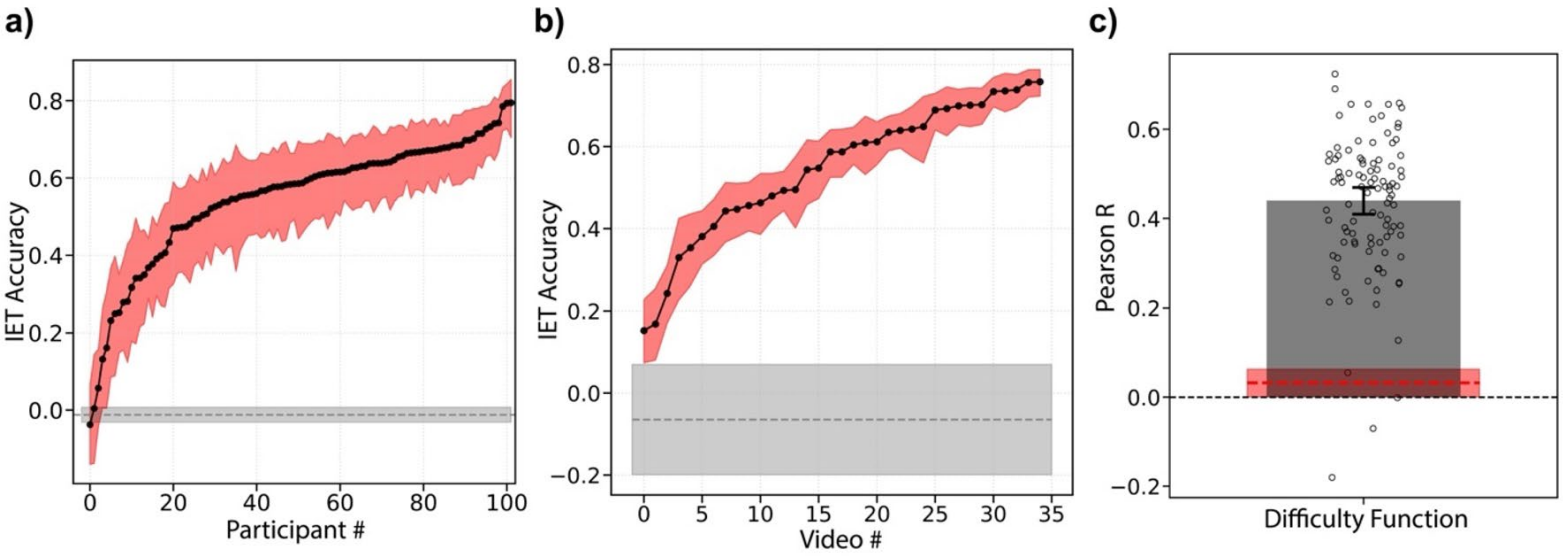
Individual differences in participant accuracy and video difficulty. (**a**) IET accuracy scores for each individual participant, ranked (**b**) IET accuracy scores for each individual video, ranked, which we call the difficulty function. Shaded red regions depict 95% bootstrapped confidence intervals for each individual participant or video. Shaded gray regions show 95% confidence intervals around the permuted null distribution; dashed grey line shows mean permuted IET accuracy. (**c**) Correlation between participants' own stimulus difficulty function and the leave-one-out group averaged difficulty function. Error bars represent bootstrapped 95% CI. Dashed red line shows bootstrapped mean permuted IET accuracy and red shaded areas show 95% confidence intervals on the permuted null distribution.

Descriptive statistics of all variables are presented in Table 1. We first quantified the individual differences in IET task accuracy, to assess whether there was systematic variability in accuracy across observers. We calculated each participant's IET accuracy for each video (for both valence and arousal ratings) and compared the average accuracy across participants (Fig. 1b; see “Methods”). IET task accuracy was calculated as the Pearson correlation between the participant’s ratings on each video and the “correct” ratings retrieved from an Informal Cultural Consensus Model^59^ (see “Methods”). The correct response computed from the Cultural Consensus Model is found by performing principal component analysis on all ratings for a given video and selecting the first set of factor scores (a weighted, linear combination of ratings)^59^. The first factor of the principal component analysis will contain individual responses that are the most correlated with each other. Essentially, the Cultural Consensus Model is a measure of the consensus judgments of valence and arousal over time for each video. It is a proxy for ground truth that is well supported for situations without an objective ground truth^59–62^. We found that the participants' IET accuracy varied significantly (Fig. 2a). We then investigated whether there were video-specific individual differences by performing a similar analysis on the accuracy of each video. Again, we found that task accuracy for each video varied significantly (Fig. 2b). To ensure that low performers in the task did not just respond randomly, we recalculated the video-specific individual differences (as shown in Fig. 2b) using a leave-one-out procedure for each participant. This allows us to organize the videos by their average accuracy across participants, which we call the difficulty function, and compare the leave-one-out group-averaged difficulty function to participants’ own difficulty functions. If each participant’s difficulty function is correlated with the leave-one-out group-averaged difficulty function, then this suggests that the participant’s accuracy on each video was correlated with the tracking difficulty of each video. That is, participants should have higher accuracy for the easier videos and lower accuracy for the harder videos. If participants have a low correlation with the group-averaged difficulty function, then this may suggest that they frequently lapsed, randomly responded, or did not actively participate in the task. We found that ~ 98% of the participants' difficulty function correlation fell outside of the permuted null distribution correlation values (Fig. 2c). This indicates that the vast majority of participants actively and consistently participated in the task. While two participants' difficulty functions fell within the 95% confidence interval of the permuted null distribution, we did not remove these subjects from the main analysis. However, in a separate analysis, we removed the two participants who fell within the permuted null distribution and found no significant difference in our results (Fig. S2).

**Table 1:** Descriptive statistics.

Variable	M	Median	SD	Min	Max	Skewness	Kurtosis
Reading the mind in the eyes	25.17	26.00	5.37	5.00	33 (91.7%)	− 1.61	3.17
Films facial expression test	27.53	28.00	3.45	10.00	32 (100%)	− 2.23	7.98
Matrices	28.61	29.00	4.39	12.00	35 (100%)	− 1.33	2.27
Vocabulary	13.17	13.00	3.60	1.00	20 (100%)	− 0.42	0.49
Age	20.15	20.00	3.00	18.00	42.00	4.62	29.06
Satisfaction w/life	23.23	24.00	6.62	5.00	35 (100%)	− 0.55	0.06
Empathy quotient	43.69	44.00	12.45	15.00	68 (85%)	− 0.34	− 0.27
Autism quotient	18.95	18.00	5.13	9.00	33 (66%)	0.46	0.11
State anxiety	42.54	42.50	12.08	20.00	70 (87.5%)	0.18	− 0.60
Trait anxiety	45.86	47.00	10.46	20.00	74 (92.5%)	− 0.15	− 0.02
Beck depression	9.64	8.00	7.89	0.00	37 (58.7%)	1.12	1.31
CAPE psychosis	72.33	71.50	15.39	46.00	126 (75%)	0.65	0.96
CAPE depressive	16.96	16.50	5.36	7.00	33 (75%)	0.37	− 0.39
CAPE negative psychosis	28.02	27.00	7.68	16.00	61 (89.7%)	1.61	4.21
CAPE positive psychosis	27.35	26.00	5.81	16.00	44 (47.8%)	0.53	− 0.24
Valence accuracy	0.63	0.68	0.19	− 0.18	0.87	− 1.85	4.12
Arousal accuracy	0.49	0.54	0.18	− 0.02	0.82	− 0.82	0.18

Percentages under Max column indicate the percentage of the max possible score for each survey obtained by our subject pool (100% indicates that the maximum possible score was obtained by our subject pool).

### Correlations between IET performance and questionnaire items

**Figure 3:**
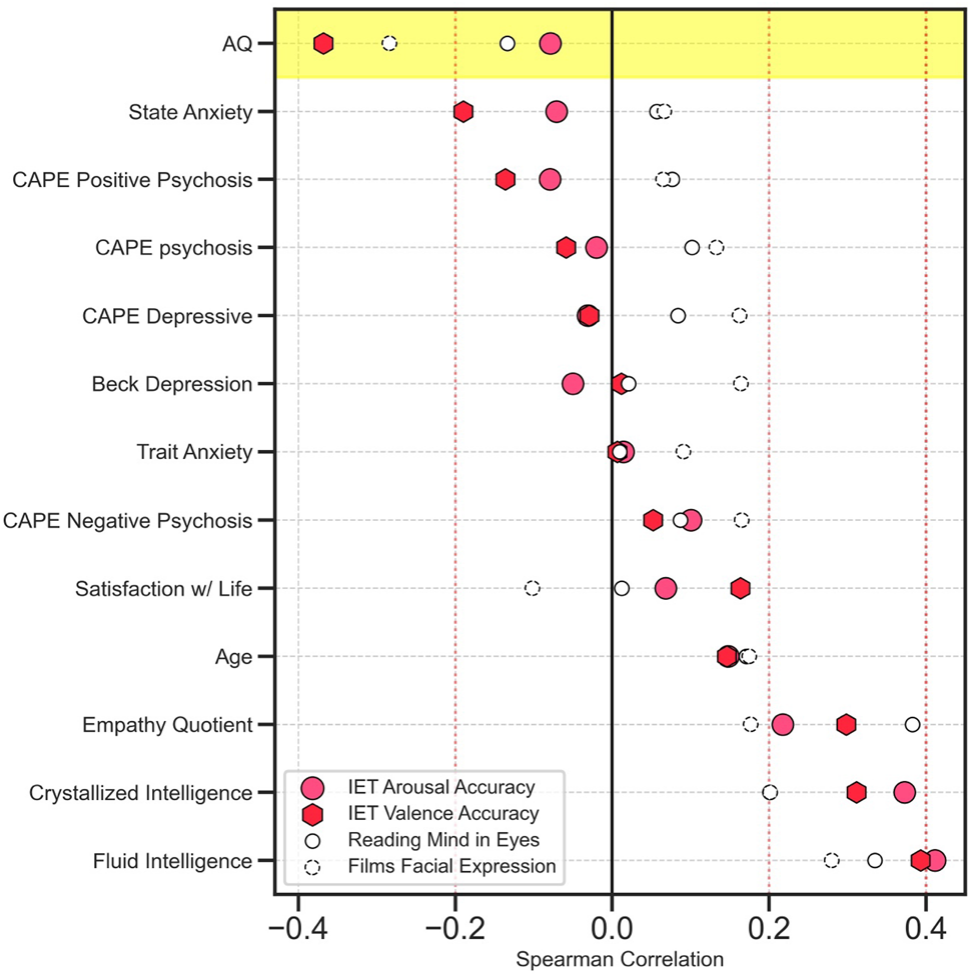
Correlations between IET and questionnaires. Correlations between IET valence (red hexagon) and arousal (pink circle) accuracy scores, the Eyes Test (solid circle), Films Facial Expression Task (dashed circle), and questionnaires completed by participants. Highlighted row shows the correlation between each task and Autism Quotient (AQ) scores.

Our main goal was to investigate whether low accuracy on the IET task was correlated with high scores on the AQ in order to explore whether individuals with ASD have impaired context-based emotion processing. We also wanted to compare this relationship to that of other popular emotion perception tasks: the Eyes Test^6^ and the Films Facial Expression Task^63^. We calculated the Spearman correlation between all variables in our data (Fig. S1), instead of the Pearson correlation to avoid any assumptions about the distribution of the data. We report both uncorrected and Bonferroni corrected significance (for 17 comparisons made in the main results; Fig. 3). We found a significant negative correlation between participants' accuracy on the IET task for their valence ratings and their AQ scores (rho = − 0.368, *p* = 0.002, Bonferroni corrected;* p* < 0.001, uncorrected). Negative, but non-significant, correlations were found for the Films Facial Expression Task and AQ (rho = − 0.284, *p* = 0.065, Bonferroni corrected;* p* = 0.004, uncorrected), the Eyes Test and AQ (rho = − 0.134, *p* = 0.180, uncorrected) and IET arousal accuracy and AQ (rho = − 0.079 *p* = 0.431, uncorrected) (Fig. 3). Significant positive correlations were also found between IET valence accuracy and the Empathy Quotient (rho = 0.298 *p* = 0.04, Bonferroni corrected; *p* = 0.002, uncorrected) and between the Eyes Test and the Empathy Quotient (rho = 0.383, *p* < 0.001; Bonferroni corrected; *p* < 0.001, uncorrected). We found no significant correlations between Films Facial Expression Task and the Empathy Quotient (rho = 0.176, *p* = 0.076, uncorrected) or IET arousal accuracy and the Empathy Quotient (rho = 0.217, *p* = 0.478, Bonferroni corrected; *p* = 0.028, uncorrected). Significant correlations were also found between Fluid Intelligence and IET valence accuracy (rho = 0.393, *p* < 0.001, Bonferroni corrected; *p* < 0.001, uncorrected), IET arousal accuracy, (rho = 0.411, *p* < 0.001, Bonferroni corrected; *p* < 0.001, uncorrected), the Eyes Test (rho = 0.335, *p* = 0.01, Bonferroni corrected; *p* < 0.001, uncorrected), but no significant correlation was found between Fluid Intelligence and Films Facial Expression Task (rho = 0.28, *p* = 0.075, Bonferroni corrected; *p* = 0.004, uncorrected). Significant correlations were also found between Crystallized Intelligence and IET valence accuracy (rho = 0.311, *p* = 0.025, Bonferroni corrected; *p* < 0.001, uncorrected) and IET arousal accuracy, (rho = 0.373, *p* = 0.002, Bonferroni corrected; *p* < 0.001, uncorrected), however, no significant correlation was present between Crystallized Intelligence and the Eyes Test (rho = 0.201, *p* = 0.733, Bonferroni corrected; *p* = 0.043, uncorrected) and Films Facial Expression Task (rho = 0.201, *p* = 0.723, Bonferroni corrected; *p* = 0. 043, uncorrected). We also recalculated the correlation between IET valence accuracy and AQ while removing the two subjects who fell within the permuted null in Fig. 2c and found that the correlation remained significant (rho = − 0.361, *p* = 0.004, Bonferroni corrected; *p* < 0.001, uncorrected).

### Controlling for general intelligence and covarying factors

**Figure 4:**
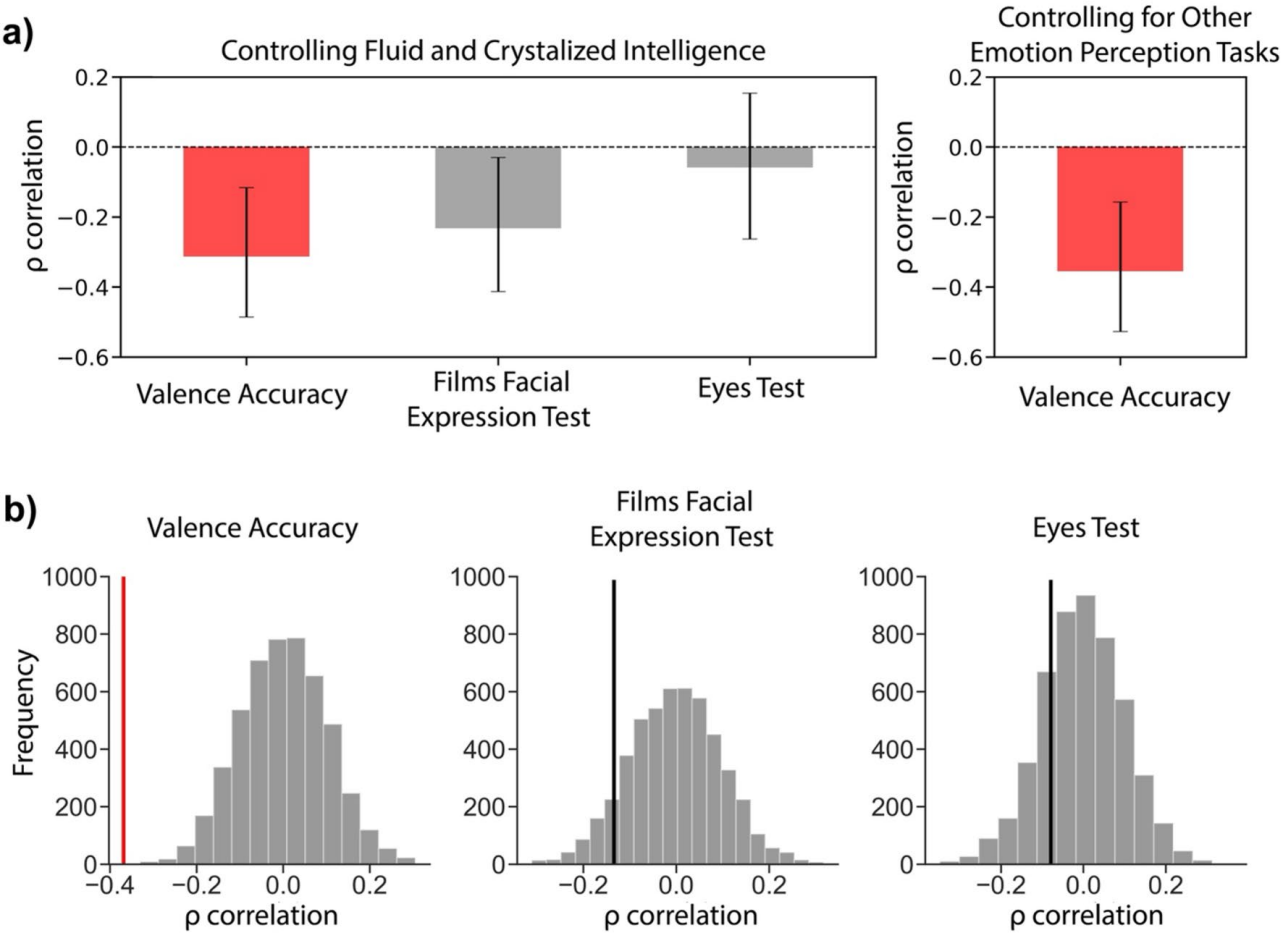
Significance tests for AQ correlations. (**a**) Partial correlations between AQ and IET valence accuracy, Films Facial Expression Task, and the Eyes Test while controlling for Fluid and Crystalized intelligence (left). We also computed partial correlations for AQ and IET valence accuracy while controlling for both the Eyes Test and Films Facial Expression Task performance (right). Error bars represent bootstrapped 95% CI. (**b**) Permutation tests for AQ correlations showing Valence accuracy, Films Facial Expression Task, and the Eyes Test from left to right. Gray distributions represent the permuted null distributions for each relationship. The solid vertical lines (red, black) represent the observed empirical correlations for each task, respectively.

We further investigated the correlation between IET valence accuracy and AQ by controlling for Fluid and Crystallized Intelligence. This assures that the correlation between the two variables is not just driven by general intelligence. We computed partial correlations between IET valence accuracy and AQ while controlling for Fluid and Crystalized Intelligence and plotted the bootstrapped mean and 95% confidence intervals revealing that the correlation does not cross 0 and remains significant (*m* = − 0.311, CI [− 0.485, − 0.117], 5000 iterations) (Fig. 4a). The correlation between Films Facial Expression Task and AQ also remained significant when controlling for general intelligence (*m* = -0.232, CI [− 0.413, − 0.03], 5000 iterations). However, the correlation between the Eyes Test and AQ was not significant (*m* = − 0.058, CI [− 0.263, 0.154], 5000 iterations). We also calculated partial correlations for IET valence accuracy and AQ while controlling for both Films Facial Expression Task and the Eyes Test performance, which revealed that the correlation remained significant (*m* = − 0.355, CI [− 0.527, − 0.157], 5000 iterations). This suggests that the correlation between IET valence accuracy and AQ is not explained by participants’ emotion perception abilities as measured by other popular face recognition tests.

In order to control for potential covarying factors, we performed new partial correlations between all tasks and AQ while controlling for general intelligence and the Empathy Quotient which had significant correlations with IET valence accuracy and the Eyes Test. We found that the correlation between IET valence accuracy and AQ remained significant (*m* = − 0.26, CI [− 0.447, − 0.057], 5000 iterations), and so did the correlation between Films Facial Expression Task and AQ (*m* = − 0.21, CI [− 0.4, − 0.003], 5000 iterations) (Fig. S3). The correlation between the Eyes Test and AQ was not significant when controlling for potential covarying factors (*m* = 0.05, CI [− 0.15, 0.25], 5000 iterations) (Fig. S3a). We also computed partial correlations while controlling for both IET valence and arousal accuracy between AQ and the emotion perception tasks. We found no significant correlation between AQ and the Films Facial Expression Task (*m* = − 0.17, CI [− 0.37, 0.04], 5000 iterations) and the Eyes Test (*m* = − 0.10, CI [− 0.29, 0.01], 5000 iterations) (Fig. S3b). This indicates that these popular tests of face emotion recognition do not account for significant variance in AQ once IET accuracy is controlled. Permutation tests were also conducted as additional statistical tests and revealed significant correlations between IET valence accuracy and AQ (*p* = 0.001, permutation test) and Films Facial Expression Task and AQ (*p* = 0.023, permutation test) (Fig. 4b). The correlation between the Eyes Test and AQ was not significant (*p* = 0.174, permutation test).

### Video analysis: isolating the best videos for predicting AQ

**Figure 5:**
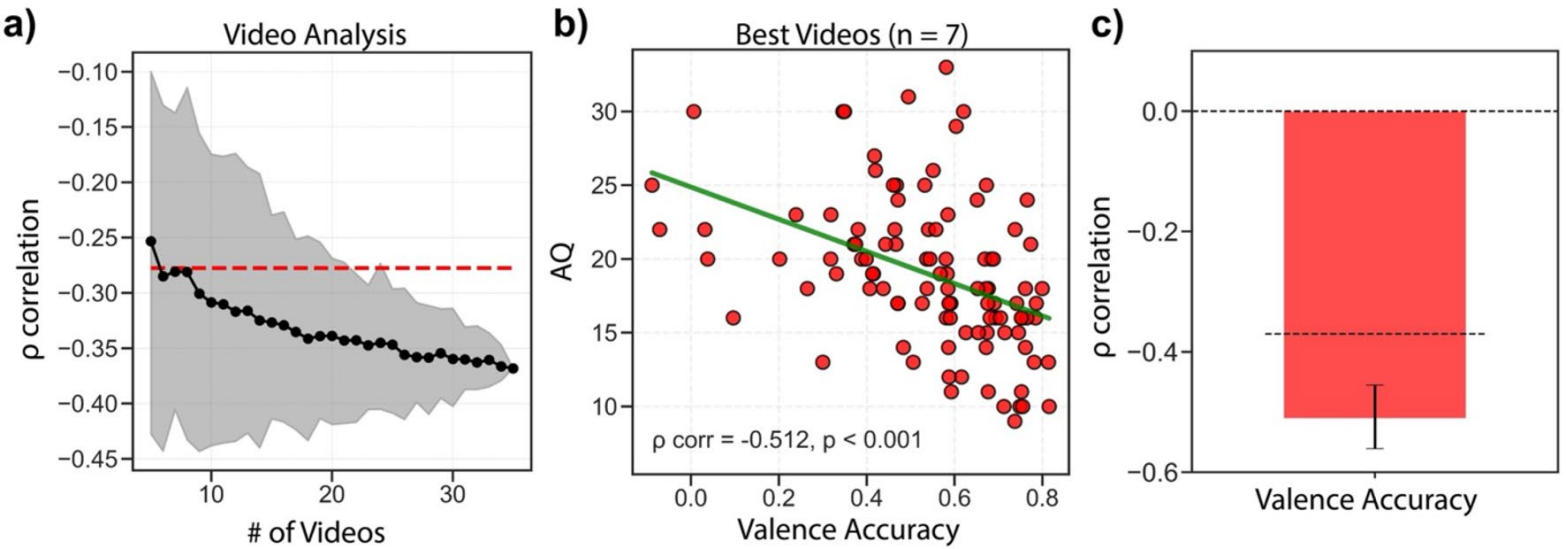
Isolating the best videos for predicting AQ. (**a**) Example of the video analysis, showing only 100 iterations for each step. In a Monte Carlo simulation, we randomly selected N videos (abscissa) from the 35 videos and bootstrapped the correlation between IET valence accuracy and AQ (ordinate). The black dots represent the bootstrapped mean Spearman correlation for each number of videos used. The red dashed line represents 75% of the size of the original effect size observed with all the videos. Only 7 videos were needed to achieve an average effect size of 75% of the original correlation. (**b**) Correlation between the 7 best videos identified from the analysis in (**a**) and AQ scores. Green solid line represents the fitted linear regression model. (**c**) Cross-validated correlation between IET valence accuracy and AQ. The data were split into 5 close-to-equal chunks and the correlation between IET valence accuracy and AQ was calculated for each chunk then averaged and was calculated for 5000 iterations. Dashed black line represents the originally observed correlation with all videos used. Error bars represent bootstrapped 95% CI.

Our next goal was to investigate which videos in the task were the best videos for assessing the relationship between IET and ASD. The original set of videos in this experiment were from a previous study^11^, and were not chosen to specifically investigate traits associated with ASD. Nevertheless, it is interesting that the correlation between IET task accuracy and AQ scores was so strong. To investigate which videos were the best for assessing ASD traits, akin to an item analysis, we calculated the minimum videos needed to reach 75% of the effect size of the original *rho* = 0.37 (i.e., threshold *rho* = 0.277). We first selected 5 videos at random, without replacement, from the list of videos and used these videos to calculate all participant's IET valence accuracy. We then calculated the spearman correlation between participants' IET valence accuracy for the currently chosen videos and AQ. At each step, we increased the number of videos used to calculate IET valence accuracy. This process was repeated 5000 times for each step in the analysis and the Fisher-Z mean correlation coefficient of the 5000 iterations between IET valence accuracy and AQ was used and compared to the 75% threshold. The results show that only 7 videos were needed to reach 75% of the effect size originally observed (Fig. 5a). We chose the 7 videos with the highest correlation between IET valence accuracy and AQ for further analysis which revealed a significant negative correlation (AQ versus IET: rho = − 0.512, *p* < 0.001) (Fig. 5b). In order to verify the strength and reliability of this relationship, we conducted a reliability test: we first split the data, at random, into five chunks as evenly as possible and then recalculated the AQ correlation in each of the five chunks. We then calculated the average correlation, using fisher-Z transformation, of the five chunks and ran the same analysis for 5000 iterations. Using only the best videos, we found that the correlation remained significant and was significantly stronger than using all the videos in the original analysis (rho = 0.51, CI [− 0.45, − 0.56], p < 0.001) (Fig. 5c). This reveals that the IET task has a substantial amount of power: it only takes a few videos to reveal a strong negative relationship with AQ scores. It further supports our original findings, that individuals with ASD may have deficits in context-based emotion perception.

**Figure 6:**
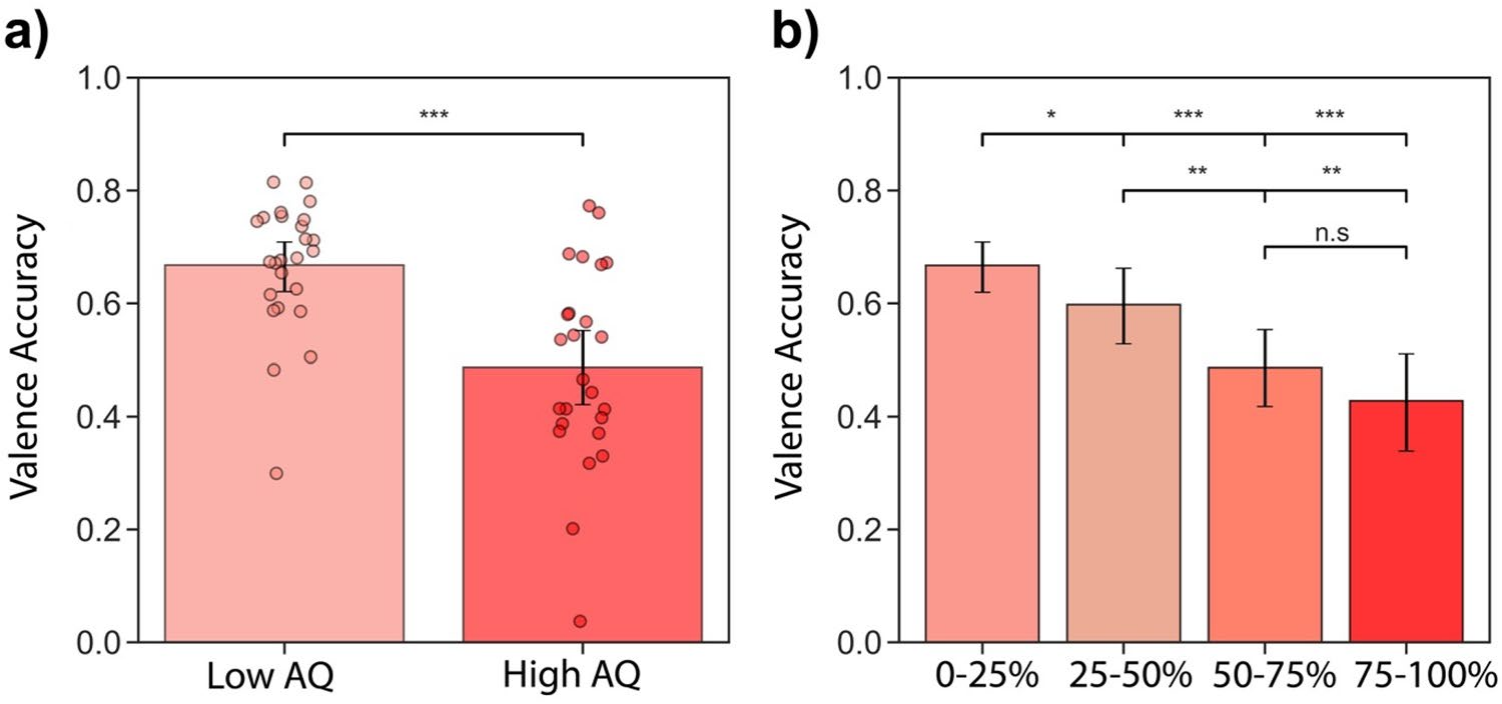
IET valence accuracy across different ranges of AQ scores for the best videos. (**a**) IET valence accuracy for low AQ scores (AQ < 18, n = 51) and high AQ scores (AQ > 18, n = 51). Each dot represents an individual participant. (**b**) IET accuracy as function of AQ quartiles: 0–25% AQ scores (9–16 AQ, n = 25), 25–50% AQ scores (16–18, AQ, n = 25), 50–75% AQ scores (18–22, AQ, n = 25), 75–100% AQ scores (22–33 AQ, n = 27). Error bars represent bootstrapped 95% CI.

Finally, we further investigated the relationship between IET accuracy and AQ for the best videos by comparing IET accuracy using a split-half analysis. We split the data into two halves using the median AQ score and categorized individuals who had an AQ score less than 18 as the “Low AQ” group and individuals who scored higher than 18 as the “High AQ” group. We found that the high AQ group had significantly lower IET accuracy than the low AQ group (Fig. 6a, p < 0.001, bootstrap test). We then wanted to explore whether the IET task is sensitive to subtle differences in AQ by splitting the data into four quartiles: the 0–25% AQ include scores 9–16 (n = 25), the 25–50% AQ includes scores 16–18 (n = 25), the 50–75% AQ include scores 18–22 (n = 25), and the 75–100% AQ includes scores 22–33 (n = 27). The 0–25% group had significantly higher IET accuracy than the 25–50% AQ group (p = 0.037, bootstrap test), the 50–75% AQ group (p < 0.001, bootstrap test) and the 75–100% AQ group (p < 0.001, bootstrap test) (Fig. 6b). The 25–50% group had significantly higher IET accuracy than the 50–75% AQ group (p = 0.017, bootstrap test), and the 75–100% AQ group (p = 0.001, bootstrap test). IET accuracy in the last two groups (50–75% and 75–100%) was not significant (p = 0.149, bootstrap test)(Fig. 6b). These results suggest that the IET task can measure subtle changes in AQ scores including in the typical range of AQ scores (9–33 score)^58^.

## Discussion

In the present study, we investigated whether context-based emotion perception is impaired in individuals who score high on the Autism Quotient (AQ), using a recently developed context-based emotion recognition task (Inferential Emotion Tracking; IET). We also compared this relationship to that of more popular assessments that use static face stimuli isolated from context. We found that participants' accuracy in IET was significantly correlated with their AQ scores, such that high AQ scores correlated with low IET task accuracy. These results indicate that context-based emotion recognition may be specifically impacted in those with Autism. Additionally, we found that the correlation between IET and AQ was stronger than the correlation between the Eyes Test and AQ, and higher than the correlation between the Films Facial Expression Task and AQ. Our results suggest that individuals with ASD may have deficits in processing emotion specifically from contextual information and they also highlight the importance of establishing ecological validity of stimuli and tasks to improve future assessments of ASD. Our result may also help explain the contradictory findings in the literature of facial emotion recognition in individuals with ASD^1^.

Whether facial emotion recognition is impaired in individuals with Autism has been under debate with some studies finding clear impairments^25,64–66^ while other studies have not^67–74^. These equivocal findings in the literature may reflect the heterogeneity of social cognition impairments in ASD, or they may be due to differences in demographic characteristics, task design (e.g. ceiling effects, variables measured, low powered studies), or task demands (e.g. context-based, dynamic, or static facial emotion recognition)^1,73^. Alternatively, this conflict may also be due to a lack of sensitivity in the behavioral measures used to assess emotion perception deficits in individuals with ASD, as studies using eye-tracking and neuroimaging methods are much more likely to find a group difference between individuals with ASD and typical controls than behavioral methods (for review, see Harms et al.^1^). Our results suggest that the inconsistent findings in the literature may be due to the lack of control or absence of contextual and dynamic information in previous studies. Moreover, our results suggest that future assessments should consider improving the ecological validity of stimuli and tasks by incorporating spatial and temporal context, thereby prioritizing the social-cognitive structure of scenes that humans typically experience in the real world^75^.

The relationship between the Eyes Tests and Autism has been extensively studied^6,7,9,76^. However, we found that participants' scores on the Eyes Test and the AQ questionnaire were not significantly correlated. This is consistent with some of the literature^18^, but may be surprising since the Eyes Test is commonly used to assess Theory of Mind in individuals with ASD and has previously been found to correlate with AQ^6^. While these results may be explained by the lack of clinically diagnosed individuals with ASD in the present study, it may also suggest that the Eyes Test is simply less sensitive: it was unable to differentiate between low and mid-range AQ scores and was not sensitive to subtle individual differences in emotion perception across participants. More popular tests used to assess ASD, like the Eye’s Test, lack both temporal and spatial contextual emotion processing which our findings reveal to be a potential core impairment in individuals with ASD. Thus, this may suggest that previous research that found no difference in performance on the Eye’s Test between healthy controls and individuals with ASD^18,77^ may be due to the lack of contextual information in the task. Additionally, low performance on the Eyes Test in individuals with ASD could reflect an impairment in facial emotion recognition due to alexithymia, which often co-occurs with ASD^78,79^.

The strength of the IET task, compared to more popular tests, is that it selectively removes the facial information of the character whose emotion is being inferred. Observers must therefore use the context to infer the emotion of the target characters. While some of the videos used in our study do include other faces, the information retrieved from these faces is not enough to accurately track the emotion of a blurred out character^11^. Consequentially, the design of the IET task and the relationship between task performance and participants’ AQ scores should not be accounted for by co-occurring alexithymia in individuals with ASD. However, we did not measure alexithymia^80^ in our subject pool and future studies should investigate whether context-based emotion perception is impaired in individuals with alexithymia.

Another strength of the IET task is that it is novel. To the best of our knowledge, only one other study has used context-only stimuli while investigating emotion recognition ability in ASD and they only used static stimuli of natural photos in their experiment^50^. Additionally, in the IET task participants must infer emotion dynamically, in real-time, meaning that they must identify changes in emotion as it occurs. This is a fundamental component of the IET task, and it reveals a potentially critical role of dynamic information in ASD. This echoes findings from previous studies, which have reported that differences in emotion recognition found in ASD may be specific to dynamic stimuli: individuals with ASD can successfully identify emotions from static images but fail to identify emotions in dynamic stimuli^53,72^. This might help explain why performance on the IET task, which requires participants to dynamically infer emotions from spatial and temporal context in real time, would have a stronger relationship with AQ than the Eyes Test and Films Facial Expression Task, both of which use static stimuli isolated from context.

Low performance on the IET task in individuals with high AQ scores may also be due to deficits in cognitive control, which is believed to be impaired in individuals with ASD^81–84^, especially when processing social stimuli^85^. Consequentially, the high cognitive demand that is required to actively infer both valence and arousal of a blurred-out character may be difficult for individuals with ASD. However, we found that IET arousal tracking did not significantly correlate with AQ scores. If a general deficit in cognitive control was driving the correlations, then we should have also found AQ scores correlated with low IET arousal tracking. It could be that individuals with higher AQ scores attended primarily to the arousal dimension instead of both dimensions, but it is not clear why this would occur consistently across individuals. Finally, low performance on the IET task may also reflect a lack of experience in social interactions in individuals with ASD. In other words, participants with high AQ scores potentially have less experience with a variety of social situations compared to participants with low AQ scores. This could interact with performance on the IET task because familiarity with a diverse range of contexts may be valuable when infering emotion in the videos.

Context-based emotion perception as a core deficit in ASD could be consistent with the weak central coherence hypothesis, which states that perception in individuals with ASD is oriented towards local properties of a stimulus and leads to impaired global processing^35,86^. Accurate perception of emotion, though, requires global processing. For example, context often disambiguates the natural ambiguity that is present in facial expressions^87^. To access this kind of global information, contextual information needs to be successfully integrated with facial information, and observers must make connections between multiple visuo-social cues across scenes and over time^11,12^. Impaired access to this global information in ASD could therefore impair emotion processing. The IET task may exacerbate the impaired central coherence in individuals with ASD, as they only have the context as a source of information when inferring the emotions of the blurred-out character in the scene. Global processing of contextual cues would be even more difficult for individuals with ASD, as they have been found to have relatively slow global processing^86,88^ and need long exposures to stimuli in order to improve global performance^89^. Thus, the dynamic nature of IET may further tax individuals with ASD, because the task not only involves spatial context (e.g., visual scene information and other faces) but also involves temporal context.

While IET valence accuracy was strongly correlated with AQ scores, IET arousal accuracy had a much weaker correlation with AQ scores (Fig. 3). The Affective Circumplex Model states that emotions can be described by a linear combination of two independent neurophysiological systems^90^; valence and arousal. Previous studies have found that the dimensional shape of valence and arousal values are constricted in individuals with ASD compared to typical controls^91^ and have found that individuals with ASD have deficits in detecting emotional valence^92–95^. Interestingly, Tseng et al. (2014)^91^ found that while children with ASD perceived a constricted range of both valence and arousal, adults with ASD perceive only a constricted range of valence, and not arousal. These findings may explain why we found that valence, and not arousal, IET tracking was negativity correlated with AQ scores. However, previous research investigating valence and arousal processing in individuals with ASD has found contradictory results^56,96^. One neuroimaging study found abnormal activation and deactivation in individuals with ASD while passively viewing dynamically changing facial expressions, suggesting that processing of valence information in individuals with ASD may be impaired^56^. However, in a more recent study, Tseng et al. investigated differences in neural activity for both valence and arousal in individuals with ASD while they actively rated the emotion of facial expressions and only group differences were found in neural activity for ratings of arousal but not for valence^96^. These contradictory results may be due to the difference in the use of static and dynamic stimuli when investigating valence and arousal perception in ASD.

While the main objective of this study was to investigate whether context-based emotion perception is impaired in individuals who score high on AQ, we also investigated its relationship with a variety of cognitive and social abilities in order to control for potential covarying factors. Other than the relationship with AQ, we also found a significant relationship between IET valence accuracy and Empathy Quotient scores. More importantly, the direction of the correlations between these surveys and IET accuracy supports previous research that has found deficits in emotional intelligence in individuals with depression^97,98^, schizophrenia^99^, and anxiety ^98^. These relationships, and all others observed in this study, suggest that IET might also be useful to evaluate an individual's emotional intelligence. IET would have great advantages in evaluating emotional intelligence as it is considered an “ability” based measure of emotional intelligence. Ability-based measures of emotional intelligence have strong advantages since the task is engaging and performance on the task cannot be faked like common-self report measures of emotional intelligence^100^. One criticism of ability-based measures is that they commonly have high correlations with general intelligence, suggesting that they may not actually be measuring emotional intelligence^101^. However, we controlled this and found that the correlation between IET valence accuracy and AQ remained significant even when general intelligence was factored out (Fig. 4a). Another criticism of ability-based measures of emotional intelligence is that they often do not correlate with outcomes that they theoretically should correlate with ^102,103^. However, we found IET accuracy for both valence and arousal to be correctly correlated with measures of depression^97,98^, schizophrenia^99^, and anxiety^98^. Consensus-based scoring has also been criticized in measures of emotional intelligence^104^, however, in our study, we use an alternative measure of consensus scoring by using Cultural Consensus Theory^59–61^. While establishing IET as a measure of emotional intelligence is beyond the scope of this study, our results hint that IET may be useful as a component of emotional intelligence metrics. This is worth investigating further in the future.

In conclusion, we investigated whether context-based emotion perception is impaired in individuals who score high on the AQ and compared this relationship with other emotion perception tasks such as the Eyes Test and Films Facial Expression Task. Our results show that performance on IET was negatively correlated with participants' AQ scores, raising the intriguing possibility that context-based emotion perception is a core deficit in ASD. Our results bring into focus a range of previous mixed findings on the relationship between emotion perception and ASD, and they shed light on possible avenues for assessing and treating ASD in future work.

## Methods

### Participants

In total, we tested 102 healthy participants (39 men and 63 women, age range 18–42, *M* = 20.19, *SD* = 2.98) on an online website created for this experiment. As a priori sample size, we aimed to collect a similar sample size as Chen and Whitney^11^ who also used the IET task in their study which had 50 participants. However, since we were interested in investigating the relationship between task performance and AQ scores, we aimed to atleast double their sample size which led to a final sample size of 102 participants. Informed consent was obtained by all participants and the study was approved by the UC Berkeley Institutional Review Board. All methods were performed in accordance with relevant guidelines and regulations of the UC Berkeley Institutional Review Board. Participants were affiliates of UC Berkeley and participated in the experiment for course credit. All participants were naive to the purpose of the study.

### Inferential Emotion Tracking

We used 35 videos used by Chen and Whitney^11^ in a previous study as stimuli for our experiment^11^ (materials available at https://osf.io/f9rxn). The videos consist of short 1–3 min clips from Hollywood movies containing single or multiple characters, home videos, and documentaries. In total, there were 25 Hollywood movies, 8 home videos, and 2 documentary clips used in the experiment. Participants used a 2D valence-arousal rating grid that was superimposed on each video clip to continuously rate the emotion of a blurred-out target character in each movie clip (Fig. 1; video shown in figure is publicly available (https://osf.io/f9rxn)). Participants were shown who the target character is before the start of the trial and were given the following instructions: “The following character will be occluded by a mask and become blurred out. Your task is to track the real emotions of this character throughout the entire video (but NOT other characters NOR the general emotion of the clip) in real-time”.

### Emotion perception tasks

Our main goal was to investigate the difference in the relationship between IET task accuracy and AQ, and the relationship between the Eyes Test and AQ. We used the revised version of the Eyes Test in this study which consisted of 36 questions where participants had to choose a mental state out of a group of words that best fit the pair of eyes shown^6^. In order to compare the results of the IET task to a general emotion perception task, we also used the Films Facial Expression Task. which investigates an individual's ability to recognize the emotional expression of others^63^. In this task, participants were presented with an adjective that represented an emotional state and participants had to select one of three images (of the same actor) that best displayed the emotional state for that trial. This task controls for general ability in recognizing emotion from facial expressions, allowing us to compare context-based emotion perception with facial expression recognition ability.

### Questionnaires

Following the completion of the IET experiment, participants were asked to complete a short (20–25 min) questionnaire. The questionnaire included a demographic section as well as a series of surveys meant to access cognitive and social ability. The first section of the questionnaire asks about gender, age, and education level. The second section contains the Satisfaction with Life Scale^105^, Autism-Spectrum Quotient^58^, Community Assessment of Psychic Experiences^106^, State-Trait Anxiety Index^107^, Beck Depression Inventory-II^108^, and the Empathy Quotient^109^. Each section is designed to assess the satisfaction with life, autism-like tendencies or characteristics, incidence of psychotic experiences, general trait anxiety, severity of depression, and ability to empathize, respectively, of the participant. Participants also completed segments from the Wechsler Adult Intelligence Scale in order to test their fluid and crystallized intelligence^110^. Specifically, we used the Vocabulary and the Matrix Reasoning subsets of the scale to measure for crystalized and fluid intelligence, respectively. These tests are well known and frequently used in psychology and have been historically used for these measures.

### Cultural consensus theory

One issue that arises with many emotion perception tasks like the Eyes Test is that there is no “correct answer” in emotion perception tasks and thus the consensus is often used as the correct answer for many emotion perception and emotional intelligence tasks^111^. For example, target words for the Eyes Test were first chosen by the authors, and a set of judges then selected which target word was the most suitable for each stimulus^6^. Five out of the eight judges needed to agree on a target word in order to label it as “correct”. One theory of consensus scoring is that the judgment of non-experts is equivalent to expert judgments except that the responses are more distributed and less reliable, thus the consensus of non-expert judgments should equal the responses of experts^112^. However, consensus scoring can be limited due to the equal weighting of participants' responses. Averaging the response of all participants assumes that all participants are equally knowledgeable, which can be invalid in emotion perception tasks^113^. In our study, we used Cultural Consensus Theory to calculate the consensus which estimates the correct answers to a series of questions by assessing an individual's knowledge or competency compared to that of the group^59,61^.

We measured accuracy on the IET task by calculating participants Cultural Consensus Theory accuracy on participants Context-only ratings. We used the Informal Cultural Consensus Model in our analysis as it makes fewer assumptions about the data and we do not need to correct for guessing^59^. Cultural Consensus Theory accuracy is calculated as the Pearson correlation between an individual observer's rating for a given video and the first set of factor scores from the principal component analysis of the Context-only ratings. We conceptualize an individual’s IET accuracy as their ability to track and infer the emotions of a blurred-out character in a movie clip by using only contextual information. We computed the average IET accuracy by first applying Fisher Z transformation on all individual correlations, averaging the transformed values, and then transforming the mean back to Pearson’s r.

## Supplementary Information

Supplementary Figures.

## Data Availability

Data are available at the OSF (https://osf.io/zku24/).

## Code availability

Data analysis was conducted using Python. Code is available at the OSF (https://osf.io/zku24/).
