# Supplementary material for: Inferential Emotion Tracking reveals impaired context-based emotion processing in individuals with high Autism Quotient scores
Source: Sci Rep. 2023 May 19;13:8093. doi: 10.1038/s41598-023-35371-6 (PMC10198990; doi:10.1038/s41598-023-35371-6)
Supplement: Supplementary file 1 — Supplementary Figures. [file 41598_2023_35371_MOESM1_ESM.docx]

| **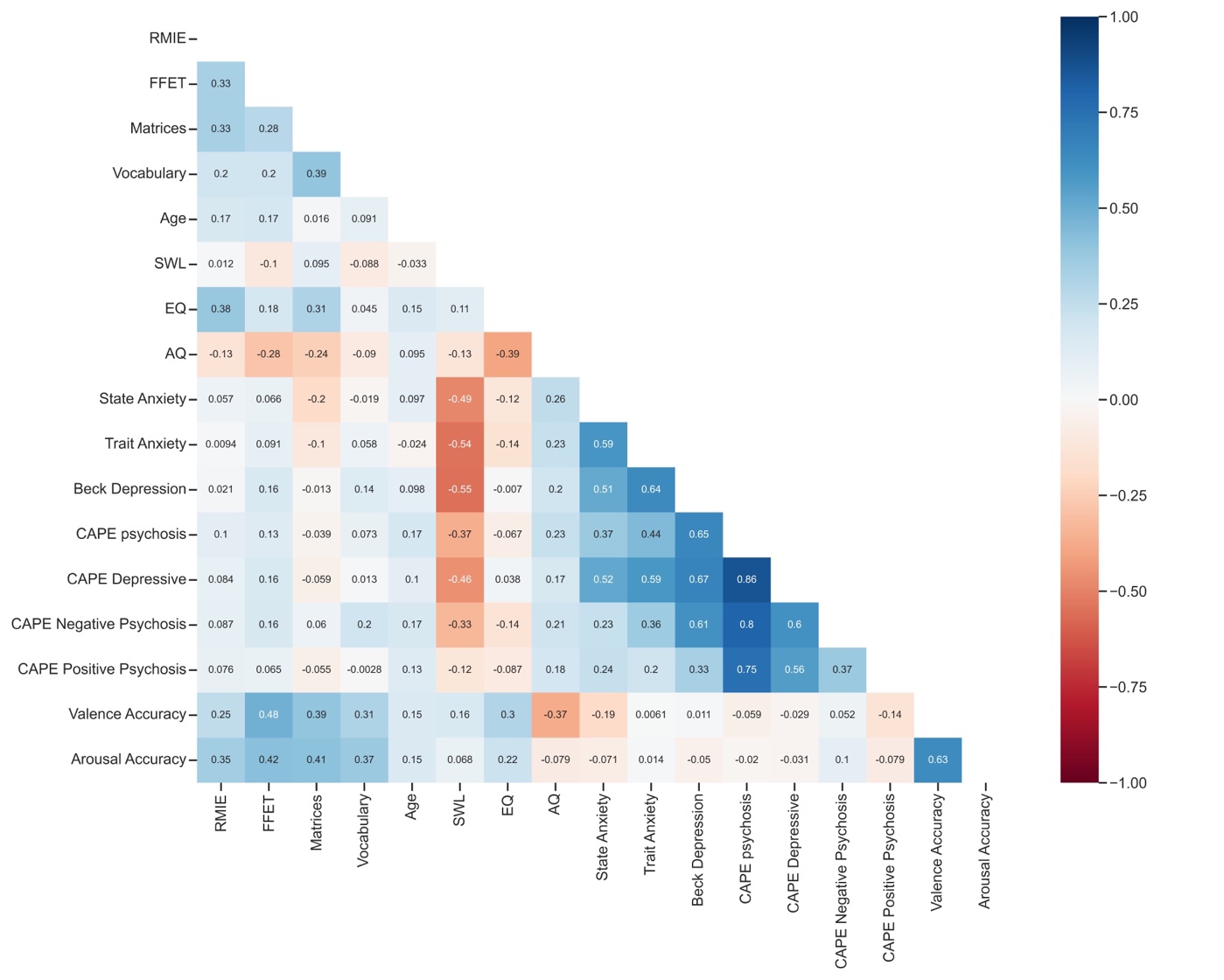** |
| --- |
| **Sup Fig. 1 - Spearman correlations between all investigated variables.** *Note:* AQ = Autism Quotient; EQ = Empathy Quotient, SWL = Satisfaction with Life, RMIE = Reading the Mind in the Eyes, FFET = Films Facial Expression Task. Self correlations are not shown. |

| **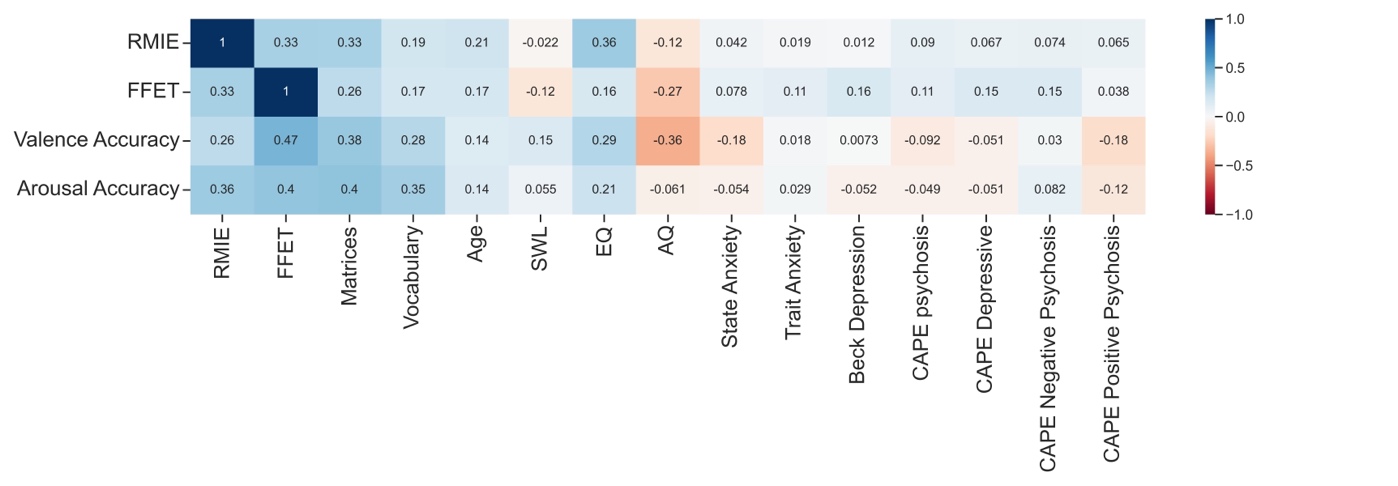** |
| --- |
| **Sup Fig. 2. Spearman correlations between all investigated variables with low performing subjects removed.** *Note:* AQ = Autism Quotient; EQ = Empathy Quotient, SWL = Satisfaction with Life, RMIE = Reading the Mind in the Eyes, FFET = Films Facial Expression Task. |

| **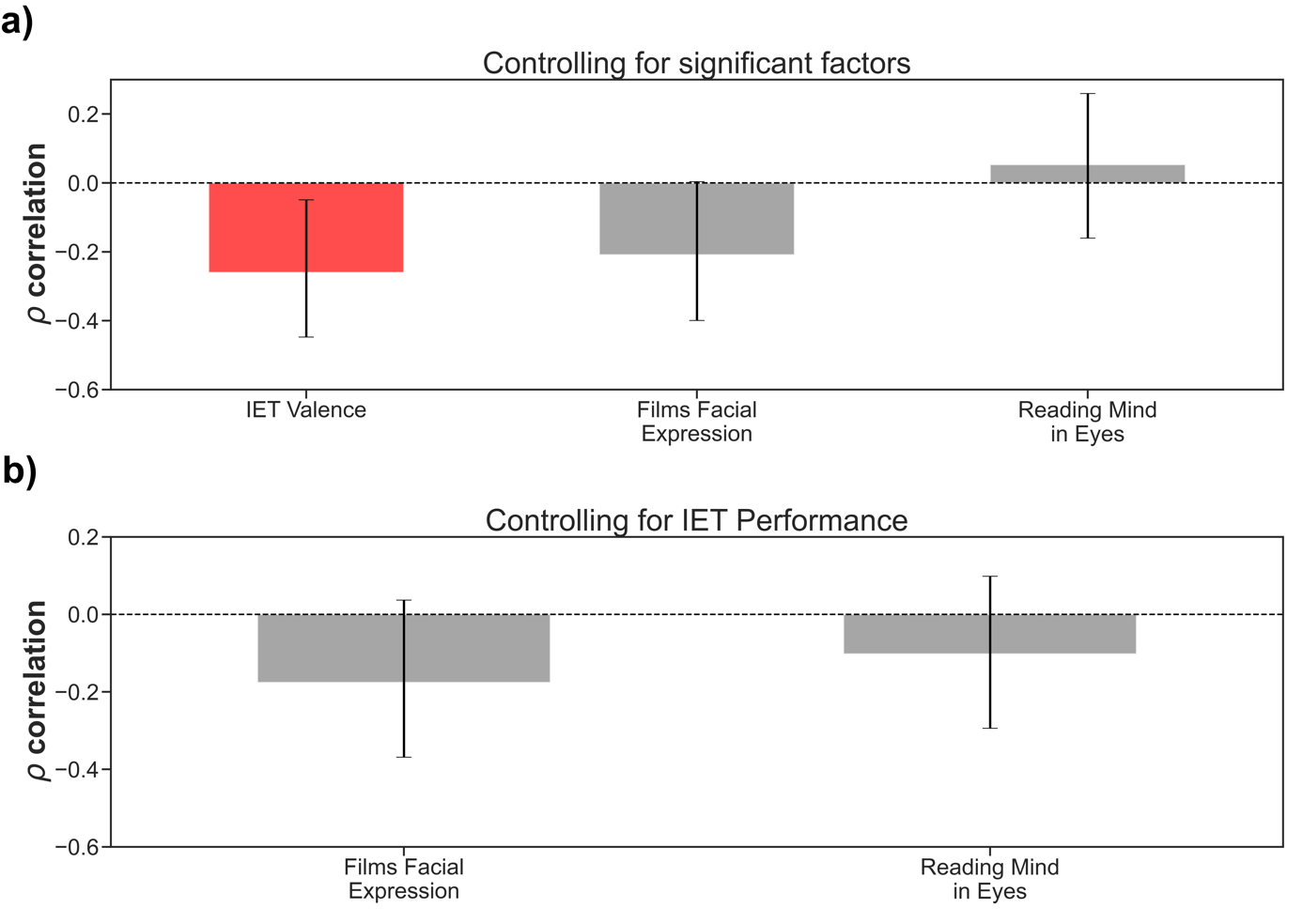** |
| --- |
| **Sup Fig. 3 – Additional partial correlations. a)** Partial correlations between AQ and IET valence accuracy, Films Facial Expression Test, and the Eyes Test while controlling for Fluid and Crystalized intelligence, and Empathy Quotient scores. Error bars represent bootstrapped 95% CI. The results indicate that IET was significantly correlated with AQ, even controlling for intelligence measures and empathy. There was no significant correlation between AQ and either the Films Facial Expression test or the Eyes Test, once intelligence and empathy were controlled. **b)** Partial correlations between AQ and, Films Facial Expression Test, and the Eyes Test while controlling for IET task accuracy. Error bars represent bootstrapped 95% CI. The result indicates that IET explains more variance in AQ scores than either of the other face recognition tests. |
